# Morbidity and Mortality Rates Following Cytoreductive Surgery Combined With Hyperthermic Intraperitoneal Chemotherapy Compared With Other High-Risk Surgical Oncology Procedures

**DOI:** 10.1001/jamanetworkopen.2018.6847

**Published:** 2019-01-11

**Authors:** Jason M. Foster, Richard Sleightholm, Asish Patel, Valerie Shostrom, Bradley Hall, Beth Neilsen, David Bartlett, Lynette Smith

**Affiliations:** 1 Department of Surgery, University of Nebraska Medical Center, Omaha; 2 Department of Biostatistics, University of Nebraska Medical Center, Omaha; 3 Department of Surgery, University of Pittsburgh Medical Center, Pittsburgh, Pennsylvania

## Abstract

**Importance:**

Currently, rates of referral of patients with peritoneal metastasis in the United States who qualify for cytoreductive surgery combined with hyperthermic intraperitoneal chemotherapy (CRS/HIPEC) are low, in part because of the misperception of high morbidity and mortality rates. However, patients requiring major gastrointestinal surgical procedures with similar complication rates are routinely referred.

**Objective:**

To evaluate the relative safety of CRS/HIPEC.

**Design, Setting, and Participants:**

Retrospective cohort study of 34 114 patients who underwent CRS/HIPEC, right lobe hepatectomy, trisegmental hepatectomy, pancreaticoduodenectomy, and esophagectomy between January 1, 2005, and December 31, 2015, included in the American College of Surgeons National Surgical Quality Improvement Project (NSQIP) database. Data analysis was performed in 2018.

**Main Outcomes and Measures:**

Data from the NSQIP database were used to compare perioperative and 30-day postoperative morbidity and mortality rates of CRS/HIPEC (1822 patients) with other, well-accepted, high-risk surgical oncology procedures: right lobe hepatectomy (5109 patients), trisegmental hepatectomy (2449 patients), pancreaticoduodenectomy (Whipple) (16 793 patients), and esophagectomy (7941 patients).

**Results:**

For 34 114 patients, median (interquartile range [IQR]) age was 63 (55-71) years and 42% were female. Patients undergoing CRS/HIPEC tended to be younger, with a median age of 57 years, and esophagectomy had the highest median (IQR) American Society of Anesthesiologists classification (3 [3-3]). When compared with CRS/HIPEC, higher complication rates were reported in the following categories: (1) superficial incisional infection in Whipple and esophagectomy (5.4% [95% CI, 4.4%-6.4%] vs 9.7% [95% CI, 9.3%-10.1%] and 7.2% [95% CI, 6.6%-7.8%], respectively; *P* < .001); (2) deep incisional infection in Whipple (1.7% [95% CI, 1.1%-2.3%] vs 2.7% [95% CI, 2.5%-2.9%]; *P* < .01); (3) organ space infection in right lobe hepatectomy (7.2% [95% CI, 6.0%-8.4%] vs 9.0% [95% CI, 8.2%-9.8%]; *P* = .02), trisegmental hepatectomy (12.4% [95% CI, 11.1%-13.7%]; *P* < .001), and Whipple (12.9% [95% CI, 12.4%-13.4%]; *P* < .001); and (4) return to the operating room for esophagectomy (6.8% [95% CI, 5.6%-8.0%] vs 14.4% [95% CI, 13.6%-15.2%]; *P* < .001). Median (IQR) length of hospital stay was lower in CRS/HIPEC (8 [5-11] days) than Whipple (10 [7-15] days) and esophagectomy (10 [8-16] days) (*P* < .001). Overall 30-day mortality was lower in CRS/HIPEC (1.1%; 95% CI, 0.6%-1.6%) compared with Whipple (2.5%; 95% CI, 2.3%-2.7%), right lobe hepatectomy (2.9%; 95% CI, 2.4%-3.4%), esophagectomy (3.0%; 95% CI, 2.6%-3.4%), and trisegmental hepatectomy (3.9%; 95% CI, 3.1%-4.7%) (*P* < .001).

**Conclusions and Relevance:**

Comparative analysis revealed CRS/HIPEC to be safe, often safer across the spectrum of NSQIP safety metrics when compared with similar-risk oncologic procedures. Patient selection was important in achieving observed outcomes. High complication rates are a misperception from early CRS/HIPEC experience and should no longer deter referral of patients to experienced centers or impede clinical trial development in the United States.

## Key Points

**Question:**

What are the morbidity and mortality of cytoreductive surgery combined with hyperthermic intraperitoneal chemotherapy (CRS/HIPEC) compared with other major oncologic surgical procedures?

**Findings:**

In this cohort study of 1822 patients who received CRS/HIPEC compared with patients who received other high-risk surgical oncology procedures, overall 30-day mortality was lower in CRS/HIPEC (1.1%) compared with pancreaticoduodenectomy (2.5%), right lobe hepatectomy (2.9%), esophagectomy (3.0%), and trisegmental hepatectomy (3.9%).

**Meaning:**

Comparative analysis revealed CRS/HIPEC to be safe across the spectrum of National Surgical Quality Improvement Project safety metrics when compared with oncologic procedures with similar inherent risk.

## Teaser

This cohort study compares outcomes of cytoreductive surgery combined with hyperthermic intraperitoneal chemotherapy for peritoneal metastasis with outcomes of similar-risk surgical oncology procedures from 2005 to 2015 using data from the American College of Surgeons National Surgical Quality Improvement Project (NSQIP).

## Introduction

The management of peritoneal metastasis continues to be one of the more challenging areas in oncology. The care of these patients requires balancing the adverse effects and tolerance of effective therapies that prolong survival and maximize quality of life, while also controlling symptoms arising from both the disease and its treatment.^1^ Worldwide, cytoreductive surgery combined with hyperthermic intraperitoneal chemotherapy (CRS/HIPEC) has emerged as one of the most important and promising therapies for improving quality of life as well as prolonging survival. Cytoreductive surgery with hyperthermic intraperitoneal chemotherapy has been the only therapy to consistently achieve significant 5-year survival rates in patients with peritoneal metastasis who have tumors arising from the appendix, colon, rectum, mesothelium, and ovary.^2,3,4,5,6,7,8,9,10^ Outside of the United States, patients with peritoneal metastasis are routinely referred for CRS/HIPEC evaluation, and this surgical therapy is considered the standard of care for patients who are candidates for optimal tumor debulking. Internationally, several clinical trials have been established not only to define the benefit of both CRS and HIPEC in patients with established peritoneal metastasis,^11^ but also to investigate the potential benefit of early HIPEC in high-risk patients without established peritoneal metastasis.^12,13^

Cytoreductive surgery is performed for peritoneal metastatic disease with the goal of removing all tumor nodules directly or with organ resection to achieve removal of all visible disease or debulk down to minimal residual disease. Hyperthermic intraperitoneal chemotherapy is the administration of heated chemotherapy at the time of surgery after completing cytoreduction. Inflow and outflow catheters are placed in the abdomen and the chemotherapy is delivered in 3 to 4 L of balanced solution. A perfusion device, similar to a heart bypass system, circulates the solution through the abdomen and heats the solution to temperatures of 40.5°C to 42.5°C. The goal is to deliver the chemotherapy and heat the peritoneal surfaces to 40.5°C to 42.5°C in effort treat any microscopic or small residual tumors to induce further cancer destruction. Hyperthermia at these temperatures administered for 90 to 120 minutes in vitro is cytotoxic to cancer cells and had yielded improved survival in peritoneal metastasis due to tumors of the appendix, colon, mesothelium, and ovary.^2,3,4,5,6,7,8,9,10,14,15^

In the United States, approximately 60 000 patients are diagnosed with peritoneal metastasis every year. However, fewer than 1000 procedures were performed in 2015. Two US randomized clinical trials were opened for colorectal metastasis and both were terminated early due to the paucity of patient accrual, which highlights the need to address CRS/HIPEC referral barriers. Several barriers exist in the United States impeding routine patient referral, as well as the development and accrual of clinical trials. Major barriers identified in the United States include lack of knowledge of the procedure, limited numbers of surgeons and centers, lack of efficacy, and safety concerns. Of these barriers, efficacy and safety concerns rank as the most significant referral deterrents, and it was reported in a recent nonpublished survey that more than 60% of referring clinicians are reluctant to send their patients owing to safety concerns. These safety concerns are a vestige of the index CRS/HIPEC studies conducted during the very first attempts of CRS/HIPEC in the 1980s and 1990s to treat peritoneal metastasis where mortality and morbidity rates approached 10% to 20% and 40% to 60%, respectively.^16,17,18^ The data reported during the first experiences do not reflect the optimization of current patient selection and improved surgical technique. Unfortunately, the stigma of these poor outcomes has remained deeply entrenched in the United States and has resulted in continued reluctance to refer patients.

Contrary to this misperception, contemporary data demonstrate mortality and morbidity rates for CRS/HIPEC of 1% to 5% and 10% to 30%, respectively.^19,20,21,22^ Specifically, these outcomes are comparable to the surgical safety of other complex oncologic abdominal procedures such as hepatectomy, pancreaticoduodenectomy (Whipple), and esophagectomy. These similar-risk complex oncologic procedures are not plagued with safety concerns impeding patient referral. Most CRS/HIPEC safety data come from noncomparative, single-center experiences, and selection bias has been another recognized limitation of these data. To best address the lack of noncomparative, multi-institutional outcome data and dispel historic misperception, a multi-institutional outcome study evaluating the relative safety of CRS/HIPEC compared with similar-risk oncologic procedures was designed. Specifically, a comparative analysis with similar-risk cancer procedures provides a context to demonstrate the current safety of CRS/HIPEC.

This study used the American College of Surgeons National Surgical Quality Improvement Project (ACS NSQIP) database to perform a comparative outcome analysis of CRS/HIPEC outcomes vs outcomes of procedures of similar inherent risk to provide context and establish the contemporary safety of CRS/HIPEC in the United States. The comparative model of similar-risk cancer operations as a frame of reference provides a platform to establish relative safety of CRS/HIPEC. The NSQIP database was selected because it is one of the largest multi-institutional outcome databases containing surgical safety data, ideal for the exploration of the hypothesis that CRS/HIPEC outcomes are comparable to those of other complex oncologic procedures.

## Methods

### Data Source and Design

The details of the ACS NSQIP, including sampling strategy, data abstraction procedures, variables collected, outcomes, and structure, are described elsewhere.^23^ We used the 2005 to 2015 ACS NSQIP Participant Use File. More than 600 universities and private medical facilities participate in ACS NSQIP and data collection. Deidentified patient information is freely available to all institutional members who comply with the NSQIP Data Use Agreement, which implements the protections afforded by the Health Insurance Portability and Accountability Act of 1996. Because the data set is deidentified by NSQIP, this investigation was exempt from review and approval as defined by the policies and procedures of the University of Nebraska institutional review board. This report follows the Strengthening the Reporting of Observational Studies in Epidemiology (STROBE) reporting guideline for cohort studies.^24^

A review of the NSQIP database (2005-2015) was performed to identify patients who underwent CRS/HIPEC, right lobe hepatectomy (RLH), trisegmental hepatectomy (TSH), Whipple, and esophagectomy using the *Current Procedural Terminology* (*CPT*) codes for each of these procedures. All procedures were performed for each year of NSQIP data collection. Any patient records that were identified as having undergone any combination of CRS, TSH, RLH, Whipple, or esophagectomy were excluded from the study. Records from the database were analyzed for rates of return to the operating room (OR), superficial incisional infection, deep incisional infection, organ space infection, median length of stay (LOS), and 30-day mortality. All *CPT* codes were cross-referenced with current *CPT* codes to ensure fidelity of recording, and these data are available on request (eTables 1 and 2 in the Supplement). The rate of comorbid factors of age, smoking, dyspnea, diabetes, weight loss, ascites, and history of congestive heart failure were also extracted from the NSQIP database.

### Statistical Analysis

All data were analyzed using PC SAS statistical software version 9.4 (SAS Institute Inc), and analyses were reported in comparison with CRS/HIPEC. Complication rates were analyzed using χ^2^ testing, and continuous variables (LOS and age) were analyzed using the Kruskal-Wallis nonparametric procedure. Both were subjected to Bonferroni corrections adjusting for multiple pairwise comparisons. A relative risk (RR) analysis was performed to more accurately quantify the risk of CRS/HIPEC compared with similar-risk procedures. The statistical level of significance was set to 2-sided .05 for all comparisons. Estimated RRs were calculated for each outcome by fitting a generalized linear model containing a term for surgical procedure using the GENMOD procedure in SAS with the binomial distribution and log link. The RR estimate, 95% confidence interval, and *P* value are presented for each procedure compared with CRS/HIPEC.

An additional generalized linear model containing terms for both surgical procedure and American Society of Anesthesiologists (ASA) classification (which measures procedures on a scale of 1-5, with 5 indicating the patient is moribund) was fit using the GENMOD procedure in SAS with a binomial distribution and log link. The RR estimates and 95% confidence intervals were compared with those from the simpler model containing surgical procedure as the only term. The results were similar, so the results from the parsimonious model are presented.

## Results

The NSQIP database query identified 34 306 patients who met inclusion criteria, and 192 records were excluded owing to overlapping surgical procedures or incompatible LOS records. The number of cases for each surgical category used in the analysis were: (1) CRS/HIPEC, 1822; (2) RLH, 5109; (3) TSH, 2449; (4) Whipple, 16 793; and (5) esophagectomy, 7941.

### Demographic Characteristics of NSQIP Surgical Categories

For 34 114 patients, median (interquartile range [IQR]) age was 63 (55-71) years and 42% were female. The median age ranged from 57 years for the CRS/HIPEC to 65 years for Whipple, and the age distribution was normal across all 5 surgical categories (eFigure 1 in the Supplement). The analysis of rates of comorbid factors (eTable 3 in the Supplement) revealed esophagectomy had the highest rates of smoking (25.3% vs 12.0%-16.1%) as well as high rates of dyspnea (10.4% vs 6.1%-6.8%) and weight loss (19.6% vs 5.6%-7.9%); Whipple had highest rates of diabetes (25.3% vs 10.4%-16.6%) and also high rates of weight loss (17.5% vs 5.1%-7.9%) and smoking (20.7% vs 12.0%-16.1%); and CRS/HIPEC had highest rates of ascites (16.1 vs 0.2%-1.3%). The esophagectomy group had the highest ASA classification risk (median [IQR], 3 [3-3]) compared with the all other groups (Table 1).

**Table 1.** American Society of Anesthesiology Classification for Each Surgical Procedure Category

Treatment	Patients, No.	% of Patients by American Society of Anesthesiologists Class^a^						
		1	2	3	4	5	1-2	3-5
CRS/HIPEC	1822	1.1	29.4	64.0	5.5	0	30.5	69.5
Trisegmental hepatectomy	2445	1.0	24.3	68.3	6.3	0.1	23.3	74.7^b^
Right lobe hepatectomy	5099	2.2	27.3	64.8	5.6	0.1	29.5	70.5
Pancreaticoduodenectomy	16 773	0.7	25.2	67.6	6.4	0.1	25.9	74.1^b^
Esophagectomy	7934	0.4	18.7	71.4	9.3	0.2	19.2	80.2^c^

Abbreviation: CRS/HIPEC, cytoreductive surgery combined with hyperthermic intraperitoneal chemotherapy.

^a^ The American Society of Anesthesiologists classifies medical risk on scale of 1 to 5 with higher scores indication increasing medical comorbidity and surgical risk.

^b^ *P *< .001 compared with CRS/HIPEC and right lobe hepatectomy.

^c^ *P *< .001 compared with all other surgical groups.

### Rates of NSQIP Morbidity, Mortality, and LOS for CRS/HIPEC Comparative Analysis

The 4 categories of surgical morbidity include 2 types of wound complications, superficial and deep, as well as intra-abdominal or organ space infection or leak and return to the OR for reexploration (Table 2). Rates of superficial infection complications were higher for Whipple (9.7%; 95% CI, 9.3%-10.1%) and esophagectomy (7.2%; 95% CI, 6.6%-7.8%) than CRS/HIPEC (5.4%; 95% CI, 4.4%-6.4%) (*P* < .001), while CRS/HIPEC superficial infection rates were comparable to those reported for RLH and TSH. Rates of deep infection were comparable for all procedures, except Whipple, for which rates were slightly higher than CRS/HIPEC (2.7% [95% CI, 2.5%-2.9%] vs 1.7% [95% CI, 1.1%-2.3%]; *P* < .01). Organ space infections occurred at higher rates for RLH (9.0%; 95% CI, 8.2%-9.8%), TSH (12.4%; 95% CI, 11.1%-13.7%), and Whipple (12.9%; 95% CI, 12.4%-13.4%) compared with CRS/HIPEC (7.2%; 95% CI, 6.0%-8.4%) (*P* = .02, *P* < .001, and *P* < .001, respectively). Rates of return to the OR were comparable to CRS/HIPEC (6.8%; 95% CI, 5.6%-8.0%) for all procedures except esophagectomy (14.4%; 95% CI, 13.6%-15.2%), for which rates were 2 times higher (*P* < .001). Median (IQR) LOS was 8 (5-11) days for CRS/HIPEC, which was lower than Whipple (10 [7-15] days) and esophagectomy (10 [8-16] days) (*P* < .001), but comparable to the other surgical categories (eFigure 2 in the Supplement). Overall 30-day mortality was 1.1% (95% CI, 0.6%-1.6%) in CRS/HIPEC, which was lower than all other groups: Whipple (2.5%; 95% CI, 2.3%-2.7%), RLH (2.9%; 95% CI, 2.4%-3.4%), esophagectomy (3.0%; 95% CI, 2.6%-3.4%), and TSH (3.9%; 95% CI, 3.1%-4.7%) (*P* < .001).

**Table 2.** Rates of Complication for CRS/HIPEC vs Other Procedures

Procedure	No. (%) [95% CI, %]					Length of Stay, Median (IQR), d
	Return to OR	Superficial Incisional Infection	Deep Incisional Infection	Organ Space Infection	Mortality	
CRS/HIPEC (n = 1822)	124 (6.8) [5.6-8.0]	98 (5.4) [4.4-6.4]	31 (1.7) [1.1-2.3]	131 (7.2) [6.0-8.4]	20 (1.1) [0.6-1.6]	8 (5-11)
Right lobe hepatectomy (n = 5109)	276 (5.4) [4.8-5.2]	235 (4.6) [4.0-5.2]	77 (1.5) [1.2-1.8]	460 (9.0) [8.2-9.8]	148 (2.9) [2.4-3.4]^a^	7 (5-9)
Trisegmental hepatectomy (n = 2449)	167 (6.8) [5.8-7.8]	162 (6.6) [5.6-7.6]	47 (1.9) [1.4-2.4]	304 (12.4) [11.1-13.7]^a^	96 (3.9) [3.1-4.7]^a^	7 (6-11)
Pancreaticoduodenectomy (n = 16 793)	1142 (6.8) [6.4-7.2]	1629 (9.7) [9.3-10.1]^a^	453 (2.7) [2.5-2.9]^b^	2166 (12.9) [12.4-13.4]^a^	420 (2.5) [2.3-2.7]^a^	10 (7-15)^a^
Esophagectomy (n = 7941)	1144 (14.4) [13.6-15.2]^a^	572 (7.2) [6.6-7.8]^b^	183 (2.3) [2.0-2.6]	604 (7.6) [7.0-8.2]	238 (3.0) [2.6-3.4]^a^	10 (8-16)^a^

Abbreviations: CRS/HIPEC, cytoreductive surgery combined with hyperthermic intraperitoneal chemotherapy; IQR, interquartile range; OR, operating room.

^a^ *P* < .001.

^b^ *P* < .01.

### Relative Risk Analysis for CRS/HIPEC Comparative Analysis

**Figure 1.**
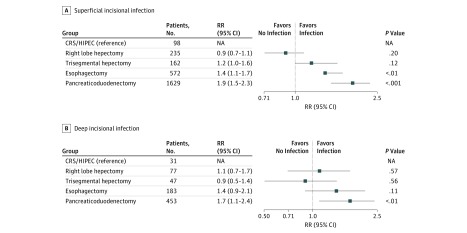
Relative Risk (RR) Analysis for Superficial and Deep Incisional Infection CRS/HIPEC indicates cytoreductive surgery combined with hyperthermic intraperitoneal chemotherapy; NA, not applicable.

**Figure 2.**
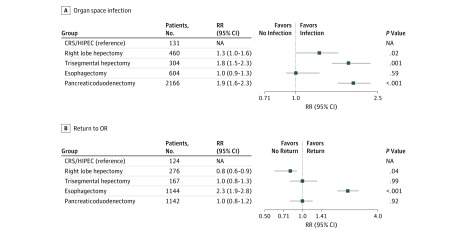
Relative Risk (RR) Analysis for Organ Space Infection and Return to Operating Room (OR) CRS/HIPEC indicates cytoreductive surgery combined with hyperthermic intraperitoneal chemotherapy; NA, not applicable.

**Figure 3.**
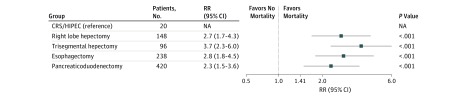
Relative Risk (RR) Analysis for Mortality CRS/HIPEC indicates cytoreductive surgery combined with hyperthermic intraperitoneal chemotherapy; NA, not applicable.

The risk of superficial incisional infection was 1.4 (95% CI, 1.1-1.8) and 1.9 (95% CI, 1.5-2.5) times greater for patients who underwent esophagectomy and Whipple than for those who underwent CRS/HIPEC (*P* < .01 and *P* < .001, respectively) (Figure 1). Deep incisional infection analysis revealed that the risk for Whipple was 1.7 (95% CI, 1.1-2.5) times higher than for CRS/HIPEC (*P* < .01) (Figure 1). Patients who underwent TSH and esophagectomy were 1.8 (95% CI, 1.4-2.4) and 1.9 (95% CI, 1.5-2.4) times more likely to experience an organ space infection compared with CRS/HIPEC (*P* < .001) (Figure 2). Patients who had esophagectomy were 2.3 (95% CI, 1.8-3.0) times more likely to experience return to the OR compared with those who had CRS/HIPEC (*P* < .001) (Figure 2). All groups had an increased 30-day mortality compared with those who underwent CRS/HIPEC: RLH (RR, 2.7; 95% CI, 1.5-4.9; *P* < .001), TSH (RR, 3.7; 95% CI, 2.0-6.8; *P* < .001), esophagectomy (RR, 2.8; 95% CI, 1.6-5.1; *P* < .001), and Whipple (RR, 2.3; 95% CI, 1.3-4.1; *P* < .001) (Figure 3). The analysis was performed with risk adjustment for ASA class, which did not affect the observed results.

## Discussion

The perception of high morbidity, high mortality, and poor surgical outcomes remains a barrier to CRS/HIPEC patient referral as well as clinical trial development in the United States, despite the published noncomparative data establishing contemporary safety. The history of CRS/HIPEC in the United States (one of the first surgical communities to attempted HIPEC) in the 1980s and 1990s was fraught with poor surgical outcomes, and the echoes of this early data continue to fuel the contemporary misperceptions in the United States regarding CRS/HIPEC surgical safety. Although abundant data have been published defining CRS/HIPEC safety,^25^ including an NSQIP series,^1,26^ these data have been criticized for being primarily single-center experiences and/or noncomparative. Reporting multicenter CRS/HIPEC outcomes in the context of similar-risk surgical procedures in a comparative model addresses the limitations of the previously published data and provides context for referring clinicians who have apprehension about CRS/HIPEC safety. In this study, CRS/HIPEC surgical safety was compared with that of similar-risk oncologic procedures in an effort to dispel historic misperceptions and provide a contemporary perspective on CRS/HIPEC safety.

The NSQIP database has become one of the most powerful resources to investigate and measure surgical safety in the United States, capturing outcome data from more than 600 US and 60 Canadian hospitals and medical centers every day. It has been integral in establishing outcome benchmarks to help individual surgeons, institutions, and surgical societies evaluate performance to help maintain and/or improve patient outcomes. The NSQIP database provides one of largest platforms to pursue noncomparative and comparative analysis with multicenter contemporary data. This comparative analysis study demonstrated not only that CRS/HIPEC was safe, but also that CRS/HIPEC had lower overall morbidity and an observed 2- to 3-fold lower mortality than Whipple, hepatectomy, and esophagectomy procedures.

### Limitations

Age and medical comorbidity are well-established variables influencing patient outcomes. The demographic analysis revealed that patients who underwent CRS/HIPEC had the lowest median age; however, the age distribution was normal across surgical categories and did not appear to be a major factor. Analysis of comorbidity rates revealed that the esophagectomy and Whipple surgical groups both had the highest rates of smoking and weight loss, while the highest rate of diabetes was in Whipple, and the highest rate of dyspnea in esophagectomy. The ASA classification comparison revealed that esophagectomy also had a statistically significant composition of higher-risk surgical patients. These factors may have influenced the observed outcome differences; however, these comorbidity factors may be inherent to the surgical populations and not amenable to further reductions in the surgical selection of patients. Additionally, the presence of these comorbidities identifies an association, but what is unknown is the medical control of modifiable factors such as glucose levels, nutritional optimization, and smoking cessation, which was beyond the scope of this analysis. These factors may have influenced the higher morbidity and mortality observed in the groups other than CRS/HIPEC and are an important area for future comparative studies.

While NSQIP provides a large database repository to perform this comparative analysis, there are other limitations in addition to medical comorbidities. The CRS/HIPEC procedure is performed for a variety of tumor histologies, the most common including appendix, colorectal, ovarian, and mesothelioma. This analysis is limited because it cannot address how each tumor type may influence the observed surgical safety. In addition, the magnitude of the surgical resections performed for the CRS/HIPEC group cannot be determined because detailed operative data were not available. Specific surgical detail, including reoperative surgery vs primary surgery, location of tumor removed, adjacent structures that required dissection, resection vs tumor ablation, number of tumors resected, and the total number of organs resected, all affect outcome but are outside the scope of this study. While CPT codes alone do not define the magnitude or level of risk of the procedures performed with HIPEC, they provide some descriptive data defining the types of procedures performed. Another major limitation is that *CPT* codes for additional procedures were not routinely reported in NSQIP. However, from the *CPT* codes available in NSQIP, 76% of patients in the HIPEC group had at least 1 non-HIPEC code identified and 46% had 2 or more codes reported (eTable 2 in the Supplement). The most common codes identified included tumor resection/ablation (30%), bowel resection (31%), and other gastrointestinal resection (28%) (eTable 1 in the Supplement).

Patient selection is an important component in all surgical procedures and, given the inherent magnitude of CRS/HIPEC, may have been more rigorous in this group. One tool used to select patients is assessment of disease burden by peritoneal carcinoma index, which quantifies the peritoneal disease burden on imaging and at the time of surgery. In general, for patients with colorectal, ovarian, high-grade appendix, and mesothelial tumors, selecting patients with a peritoneal carcinoma index score of less than 25 is important in achieving long-term survival.^27,28,29,30,31,32^ This also enables surgeons performing CRS/HIPEC to achieve optimal surgical cytoreduction, which is paramount in and predictive of long-term survival.^29^ Additionally, surgeons who perform CRS/HIPEC in the United States are a small community who primarily perform these procedures at high-volume centers because only a limited number of institutions and medical centers are equipped or willing to invest in a CRS/HIPEC program. In the United States, there are approximately 20 high-volume centers and 90% of them are NSQIP participating centers. The observed outcomes in this study may have been indirectly affected by high-volume centers’ experience factor compared with the other surgical categories, resulting in the observed lower CRS/HIPEC mortality and complication. Most importantly, these data establish a high quality benchmark for CRS/HIPEC outcomes derived from a robust multicenter surgical experience.

While these data address the surgical safety of CRS/HIPEC, they do not provide perspective on the impact of the oncologic survival benefit of CRS/HIPEC in treating peritoneal metastasis, which is equally important in defining the value of this procedure and has been a referral barrier. In patients with colorectal peritoneal metastasis treated with chemotherapy alone, median overall survival ranges from 7.8 to 15.2 months, while in patients treated with CRS/HIPEC, median survival ranges from 22 to 47 months, with 5-year survival of 27% to 54%.^33^ The most recent randomized phase 3 trial in colorectal peritoneal metastasis, PRODIGE 7,^29^ reported a remarkable 40-month median survival for patients treated with CRS with or without HIPEC and identified the group with modest tumor burden (peritoneal carcinoma index score of 5-15) who may benefit from the addition of 30 minutes of HIPEC with oxaliplatin. In mesothelioma and appendix peritoneal metastasis, CRS/HIPEC has significantly improved 5-year survival from less than 10% to 50% to 90%, and CRS/HIPEC is considered the standard of care for these tumor types.^10,31,34,35,36,37,38^ Data continue to emerge that support adding HIPEC to CRS surgery in ovarian cancer, with the largest series (1051 patients) receiving CRS/HIPEC reporting a median survival of 73 months.^30^ Also, 2 recent phase 3 randomized clinical trials investigating CRS/HIPEC in recurrent and primary stage III ovarian cancer demonstrated that HIPEC yielded a 2-fold overall survival benefit and 11-month overall survival benefit, respectively.^2,32^ Comparatively, from the perspective of relative oncologic survival benefit for surgical procedures of similar risk, Whipple has a 5-year survival rate of 15% to 25%^39^; hepatectomy for colorectal cancer, 35% to 50%^40,41^; and esophagectomy, 30% to 60%.^42,43^ Based on these current data, CRS/HIPEC also has an oncologic survival benefit similar to these procedures.

In summary, this analysis revealed that patients undergoing CRS/HIPEC have shorter LOS, lower morbidity rates, and lower mortality rates when compared with patients undergoing similar-risk oncologic procedures. In a comparative NSQIP analysis, CRS/HIPEC was performed with a high level of safety, providing evidence to dispel misperceptions rooted in the historic data. Patient selection was an important component in achieving the observed outcomes. Safety concerns should no longer be a deterrent to routine referral to high-volume centers. Conducting CRS/HIPEC clinical trials in the United States is the next step to improving patient selection and HIPEC agents, optimizing HIPEC duration, and identifying biological mechanisms and pathways involved in responsive and nonresponsive peritoneal surface disease. Through increased use and clinical trial development of CRS/HIPEC, there is a great opportunity to improve outcomes in peritoneal metastasis.

## Conclusions

Patients experiencing peritoneal, liver, pancreatic, and esophageal cancers, whether primary or metastatic, face a negative prognosis in the absence of treatment. Surgical treatment has long been established as one of the successful treatments available for these cancers; therefore, treating physicians commonly refer patients, except for those with peritoneal metastasis. A major deterrent to refer patients with peritoneal metastasis for CRS/HIPEC is surgical safety, which, historically, was a concern. Although contemporary safety of CRS/HIPEC has improved greatly, physician referral has not increased owing to the lack of dissemination and demonstration of the contemporary safety of CRS/HIPEC.

A comparative analysis using the NSQIP database to establish the safety of CRS/HIPEC revealed that CRS/HIPEC is not only safe when compared with procedures of similar risk, but is often associated with less morbidity. This study found that CRS/HIPEC had the lowest mortality risk, almost 50% to 75% lower than other advanced oncology surgical procedures. Patient selection is an integral part in surgical outcomes in patients undergoing CRS/HIPEC. These findings provide objective data to dispel the misperception of morbidity and mortality concerns surrounding CRS/HIPEC, and surgical risk should no longer remain a deterrent to patient referral or development of clinical trials for CRS/HIPEC.

## Notes

Supplement. **eFigure 1.** Age Distribution for Each Treatment **eFigure 2.** Length of Stay Duration for Each Treatment **eTable 1.** CPT Codes Utilized **eTable 2.** Frequency of Multiple CPT Codes **eTable 3.** Rates of Comorbidities
