# Supplementary material for: Morbidity and Mortality Rates Following Cytoreductive Surgery Combined With Hyperthermic Intraperitoneal Chemotherapy Compared With Other High-Risk Surgical Oncology Procedures
Source: JAMA Netw Open. 2019 Jan 11;2(1):e186847. doi: 10.1001/jamanetworkopen.2018.6847 (PMC6484874; doi:10.1001/jamanetworkopen.2018.6847)
Supplement: Supplement. — eFigure 1. Age Distribution for Each Treatment eFigure 2. Length of Stay Duration for Each Treatment eTable 1. CPT Codes Utilized eTable 2. Frequency of Multiple CPT Codes eTable 3. Rates of Comorbidities [file jamanetwopen-2-e186847-s001.pdf]

Supplementary Online Content

Foster JM, Sleightholm R, Patel A, et al. Morbidity and mortality rates following cytoreductive surgery combined with hyperthermic intraperitoneal chemotherapy compared with other high-risk surgical oncology procedures. *JAMA Netw Open*. 2019;2(1):e186847. doi:10.1001/jamanetworkopen.2018.6847

eFigure 1. Age Distribution for Each Treatment

eFigure 2. Length of Stay Duration for Each Treatment

eTable 1. CPT Codes Utilized

eTable 2. Frequency of Multiple CPT Codes

eTable 3. Rates of Comorbidities

This supplementary material has been provided by the authors to give readers additional information about their work.

© 2019 Foster JM et al. *JAMA Network Open.*

eFigure 1

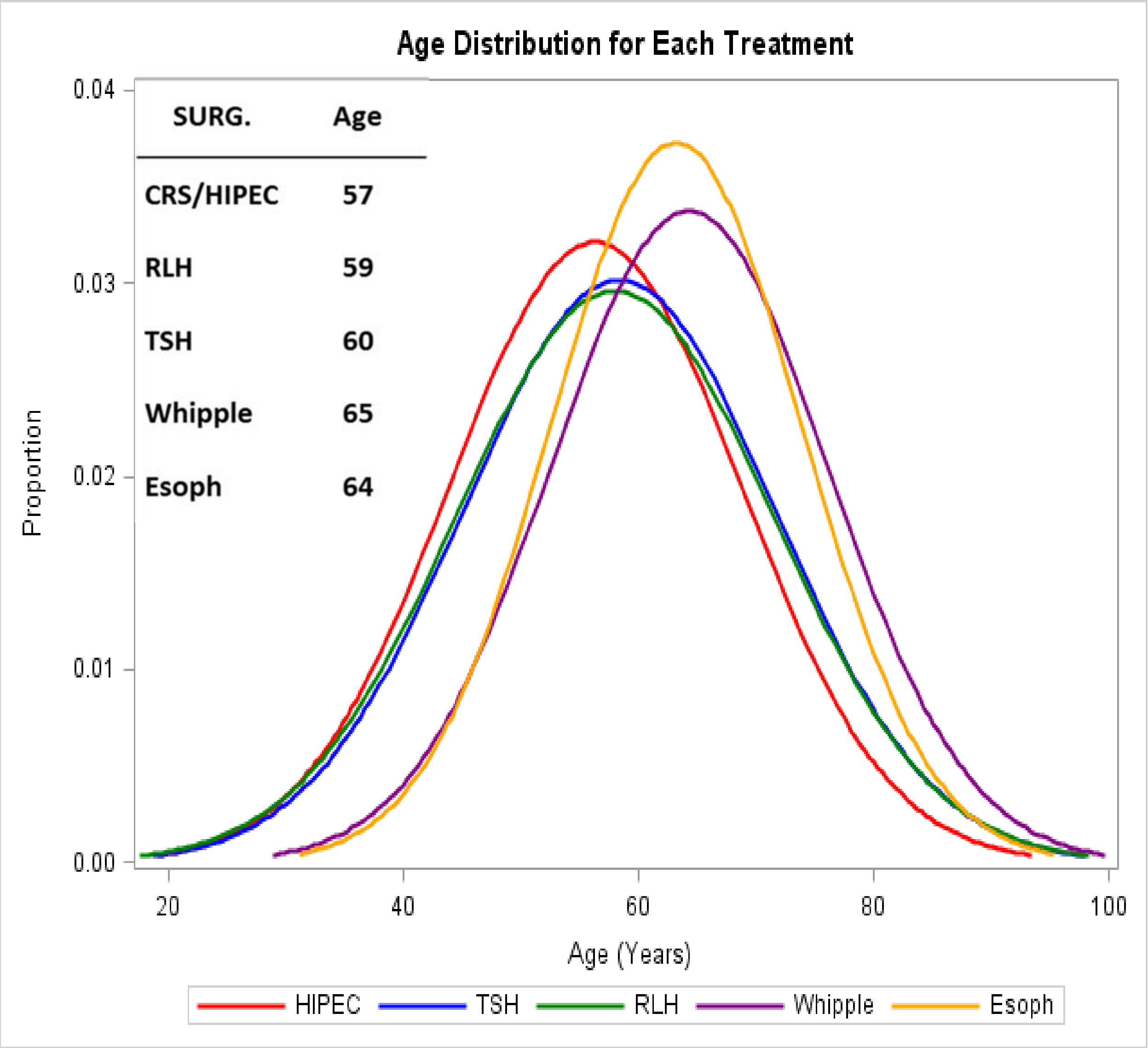

© 2019 Foster JM et al. *JAMA Network Open.*

eFigure 2

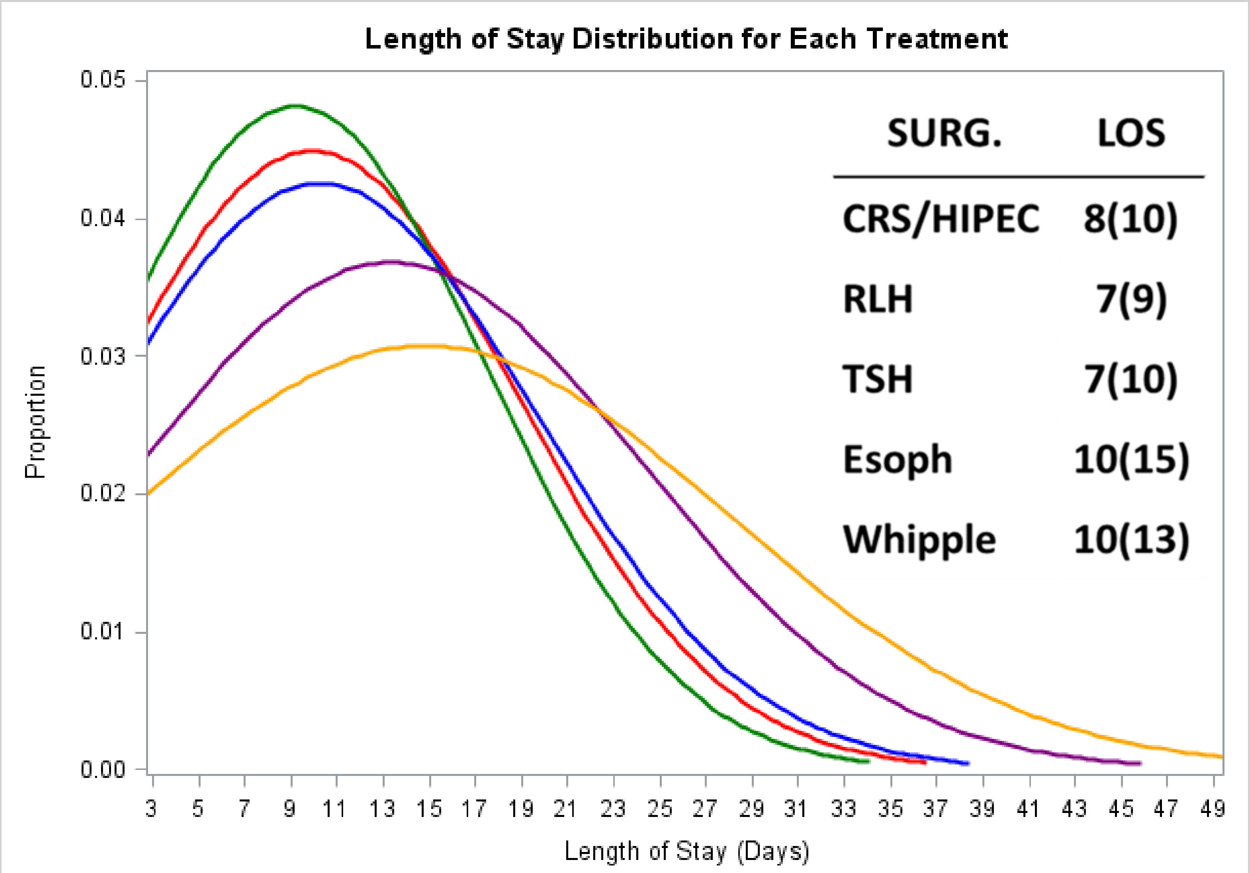

© 2019 Foster JM et al. *JAMA Network Open.*

eTable 1 CPT Codes utilized

**cpt CODES**			
Tumor resection	49205	203	11%
	49204	75	4%
	49203	265	15%
**TOTAL**		**543**	**30%**
Small or Large intestine	44150	26	1%
	44140	204	11%
	44145	37	2%
	44160	128	7%
	44120	176	10%
**TOTAL**		**571**	**31%**
**Other GI resections**			
Distal panc	48140	66	4%
Splenectomy	38100	82	5%
Choly	47600	327	18%
	58180	31	2%
**TOTAL**		**506**	**28%**
Other cpt codes	see below	1178	65%

Other cpt codes: 49180, 49185, 49215, 49220, 49250, 49255, 58953, 58954, 58720, 58150

© 2019 Foster JM et al. *JAMA Network Open.*

eTable 2 Frequency of multiple CPT codes

# of codes	Frequency	Percent	Cumulative Frequency	Cumulative Percent
0	441	24.2	471	24.2
1	551	30.22	1059	54.42
2	419	23.02	1507	77.44
3	257	14.13	1782	91.57
4	107	5.86	1896	97.43
5	39	2.16	1938	99.59
6	7	0.41	1822	100

© 2019 Foster JM et al. *JAMA Network Open.*

eTable 3. Rates of Comorbidities

Treatment	Age	smoke	dyspnea	diabetes	wtloss	ascites	hxchf
CRS/HIPEC	57	11.8%	6.7%	10.4%	5.6%	16.1%	0.4%
RLH	59	15.3%***	6.1%	15.6%***	5.1%	0.9%****	0.2%
TSH	60	16.1%****	6.7%	13.8%***	7.9%	1.3%****	0.3%
Whipple	65	20.7%****	6.8%	25.3%**	17.5%****	0.5%****	0.4%
Esophagectomy	64	25.3%****	10.4%****	16.6%****	19.6%****	0.2%****	0.3%
* P<0.05							
**P<0.01							
***P<0.001							
****P<0.0001							

© 2019 Foster JM et al. *JAMA Network Open.*
